# Preparation and Evaluation of a Combination of Chelating Agents for the Removal of Inhaled Uranium

**DOI:** 10.3390/molecules29235759

**Published:** 2024-12-05

**Authors:** Lintao Li, Runtian Li, Ruohan Guo, Shuang Guo, Xuan Qiao, Xinru Wu, Peng Han, Yunbo Sun, Xiaoxia Zhu, Zhuona Wu, Hui Gan, Zhiyun Meng, Guifang Dou, Ruolan Gu, Shuchen Liu

**Affiliations:** Department of Pharmaceutical Sciences, Beijing Institute of Radiation Medicine, Beijing 100850, China; li940408@126.com (L.L.); lrt870229@163.com (R.L.); guorh0217@163.com (R.G.); guos1357@163.com (S.G.); 15234444223@163.com (X.Q.); xinru_wu01@126.com (X.W.); 15901135949@163.com (P.H.); sunyunbo0919@126.com (Y.S.); 13681022512@163.com (X.Z.); wznphd@126.com (Z.W.); ganh2003@163.com (H.G.); mengzhiyun@vip.163.com (Z.M.)

**Keywords:** uranium exposure, aerosol inhalation, chelating agent, decorporation, HOPO

## Abstract

Inhalation of aerosolized uranium is recognized as a principal mode of exposure, posing significant risks of damage to the lungs, kidneys, and other vital organs. To enhance nuclide elimination from the body, chelating agents are employed; however, single-component chelators often exhibit limited spectral activity and low effectiveness, resulting in toxicologically relevant concentrations. We have developed a composite chelating agent composed of 3,4,3-Li(1,2-HOPO), DFP, and HEDP in optimized ratios, demonstrating marked improvements in eliminating inhaled uranium. The selection of these components was initially guided by an agarose gel dynamics method, focusing on uranium binding and removal efficacy. Optimization of the formula was conducted through response surface methodology in a cellular model. The compound’s ability to enhance survival rates in mice subjected to acute uranium inhalation was confirmed, showing a dose-dependent improvement in survival in severely affected mice. Comparative assessments indicated that this multifaceted chelating agent substantially surpasses the uranium tissue clearance achieved by individual chelating agents.

## 1. Introduction

Various pathways, such as ingestion, inhalation, intraperitoneal administration, transdermal absorption, subcutaneous injection, or contamination via wounds, facilitate uranium exposure [1,2]. Workers typically encounter this hazard through airborne routes in industrial environments, whereas the general population often ingests uranium through food and water. This ingestion stems from uranium’s natural prevalence in the local hydrogeologic settings [3]. Uranium compounds inhaled exhibit diverse physical and chemical characteristics that are influenced by their type and origin. For instance, nuclear facilities utilize uranium and mixed plutonium–uranium oxide (MOX), whereas non-military applications include using depleted uranium for aircraft counterweights and radionuclide transport containers, enhancing the risk of environmental contamination [4,5,6,7,8]. Research post the 1991 Gulf War revealed that 17% to 48% of aerosolized depleted uranium from weapon detonations is soluble, with 52% to 83% remaining insoluble. The integration of uranium into the bloodstream and its eventual distribution across bodily tissues largely depends on the particulate matter’s size and solubility. The toxicological impact of uranium varies with its dose, form, exposure route, and duration, mainly manifesting in its chemical toxicity over radiological effects. Such toxicity is known to cause lung cancer and renal impairment, with severity contingent on the exposure dosage and pathway [9]. Therefore, radionuclides retained in the lungs and body after inhalation should be treated as soon as possible. Insoluble radionuclides inhaled through the respiratory tract are often washed out by whole lung lavage, and soluble radionuclides entering the blood are often removed by chelating agents.

**Figure 1:**
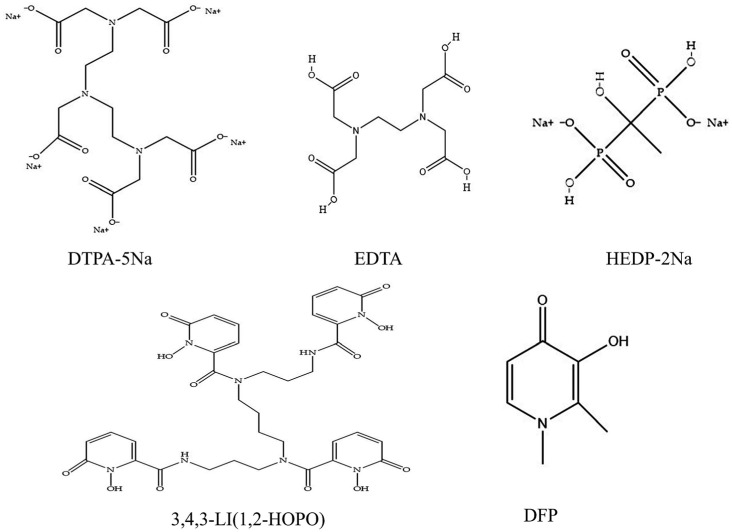
Some of the chelating ligands’ structures are shown in the figure.

Chelators are a category of molecules that bind metal ions, forming stable complexes that facilitate the removal of heavy metals. Such chelation therapies are crucial in mitigating risks associated with radionuclide-induced diseases [10]. A variety of chelators and administration methods have been developed to enhance radionuclide clearance, addressing challenges such as low tissue specificity and high toxicity [11]. Numerous chelating ligands have been confirmed as effective for in vivo actinide complexation [12]. The main families of ligands comprise polyaminocarboxylic acids (Diethylenetriaminopentaacetic acid (DTPA); Ethylenediaminetetraacetic acid (EDTA)); Siderophores(3,4,3-LI(1,2-HOPO); DFP); macrocyclic compounds; poly-phosphonates (HEDP) [13]. Some of their structures are shown in Figure 1. These chelators demonstrate high affinity and selectivity for actinides and/or exhibit enhanced lipophilicity which enables them to penetrate biological membranes. Some of these chelating agents have already been approved as clinical drugs or are in the preclinical stages, which further highlights their potential value and importance in the medical field. For example: the calcium- and zinc-DTPA have received U.S. Food and Drug Administration (FDA) approval for the decorporation of plutonium, americium and curium. HEDP has been approved by the FDA in the medical treatment of bone disease [14]. 3,4,3-LI(1,2-HOPO) is recognized for its efficacy in decorporating plutonium and americium and has demonstrated capabilities for chelating uranium and Neptunium. It received Investigational New Drug (IND) designation from the U.S. Food and Drug Administration (FDA) in August 2014 and is currently awaiting Phase I clinical trials [15]. While some chelates are in clinical use, the effectiveness of single-agent chelation therapies often remains constrained by a limited chelation spectrum inherent to their singular molecular structure. Thus, formulating multiple chelators or a combination of chelators with other ligands could enhance the chelation response to multiple actinides [16]. Recent studies have incorporated bicarbonate with chelating agents to augment actinide excretion efficacy when multiple radionuclides are involved [17]. Such combination therapies could potentially improve the clearance of actinides from specific body tissues [18,19,20].

This investigation concentrated on eliminating uranium contamination from inhalation and utilized response surface methodology to develop and refine an efficacious composite chelating agent. This method was chosen to enhance the efficiency of uranium extraction from biological systems. The study employed statistical techniques to assess how variables such as the concentration and type of chelating agents affect the uranium removal process and to determine the most effective formula using mathematical modeling. Additionally, the safety and cytocompatibility of this chelating formulation were analyzed to confirm its practical applicability and effectiveness. The capability of the formulated agent was subsequently confirmed through in vivo testing on animal models, which demonstrated its potential to expedite uranium clearance and increase survival rates following severe uranium exposure.

## 2. Results

### 2.1. Comparison of Transfer Rates of Uranium Nitrate for Different Chelating Agents at Equal Concentrations with In Vitro Agarose Gel Kinetics Model

Investigating the influence of chelating agents on radionuclide extraction in vitro [21,22,23] reveals diverse absorption behaviors as defined by ICRP: rapid (type F), moderate (type M), and gradual (type S) [24,25]. Uranyl nitrate and U₃O₈ were selected to represent these varying absorption types. The dissolution of these compounds involves dynamic interactions among multiple variables, suggesting that actinides do not exist merely as simple ions at physiological pH when biological ligands are present. This complexity underscores the distinction between in vitro and in vivo experimental conditions. A biphasic acellular in vitro assay was employed, integrating static retention and dynamic transfer phases, to conduct a comparative analysis of chelator effectiveness. This approach minimizes the impact of extraneous ions on the reaction system, allowing for a precise assessment of the chelator’s complexation capacity with the nuclides.

**Figure 2:**
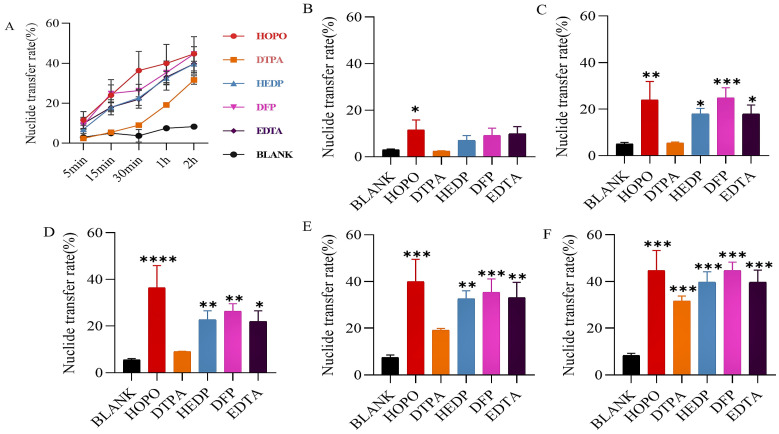
Uranyl nitrate was included in the static retention phase prepared in NS. After 5 min (**B**), 15 min (**C**), 30 min (**D**), 1 h (**E**), and 2 h (**F**) incubation containing different chelating agents, the nuclide transfer rate was measured using ICP-MS (Model 7900, Agilent, Santa Clara, CA, USA). (**A**) show the trends in nuclide transfer rates of different types of chelating agents over time. Results are expressed as the content in the mobile phase/total content in the gel × 100. * *p* < 0.05, ** *p* < 0.01, *** *p* < 0.001, **** *p* < 0.0001 is compared to the most significant group exposed to blank. Each experimental condition was performed in triplicate. Bars represent mean ± sd.

Agarose gel kinetics screening was employed to evaluate the mobilization effectiveness and transfer rates of various equal-concentration chelators on soluble uranium (uranyl nitrate) and insoluble uranium (U_3_O_8_). The study findings reveal that chelators universally enhance uranium elimination from the static phase (Figure 2A). As detailed in Figure 2B–F, HOPO and DFP have a more significant effect on accelerating U mobilization in the early stage compared to the other chelating agents when combined with the significance of the complexing ability of the chelating agent and the magnitude of the nuclide transfer rate at different time points.

**Figure 3:**
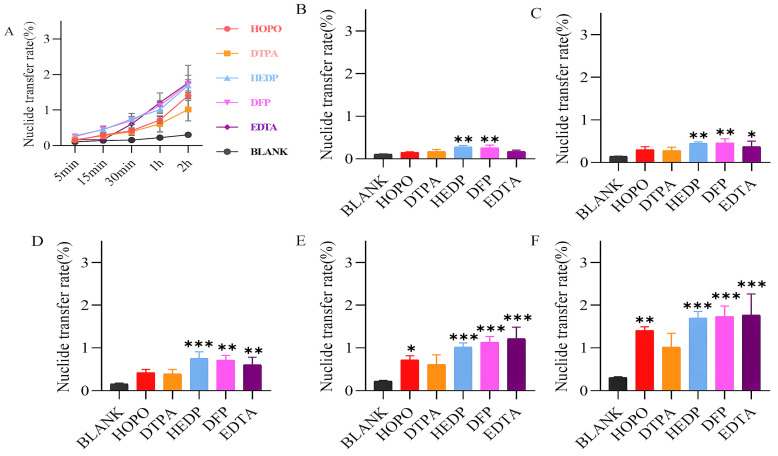
U_3_O_8_ was included in the static retention phase prepared in NS. After 5 min (**B**), 15 min (**C**), 30 min (**D**), 1 h (**E**), and 2 h (**F**) incubation containing different chelating agents, the nuclide transfer rate was measured using ICP-MS. (**A**) show the trends in nuclide transfer rates of different types of chelating agents over time. Results are expressed as the content in the mobile phase/total content in the gel × 100. * *p* < 0.05, ** *p* < 0.01, *** *p* < 0.001 is compared to the most significant group exposed to blank. Each experimental condition was performed in triplicate. Bars represent mean ± sd.

Regarding U_3_O_8_ (Figure 3), only certain chelators initiate early emission mobilization, with HEDP and DFP demonstrating superior performance (Figure 3B–F). Consequently, HOPO, DFP, and HEDP were identified for advanced formulation studies.

### 2.2. Cell Experiment

**Figure 4:**
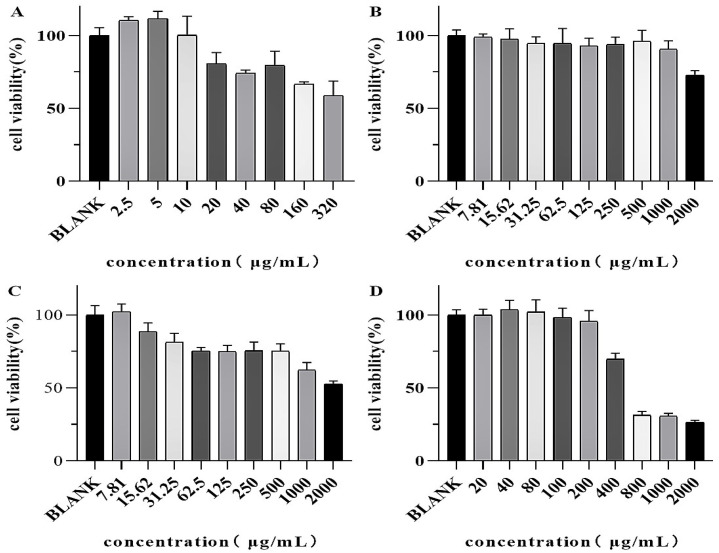
Cytotoxicity assay results. The upper limit of the dosing concentration (Uranyl nitrate (**A**), HOPO (**B**), DFP (**C**), HEDP (**D**)) was set at a cell viability rate of greater than 75%. Each concentration was repeated six times.

For the formulation studies involving the trio of chelating agents, BEAS-2B human lung epithelial cells were utilized. Prior to the main experiments, the cytotoxicity assay was conducted to establish the appropriate exposure level of uranyl nitrate at 20 µg/mL and to determine the maximum dose of each chelating agent that is tolerated HOPO ≤ 1000 μg/mL (1.332 mM), DFP ≤ 500 μg/mL (3.6 mM), and HEDP ≤ 200 μg/mL (0.877 mM). These findings are depicted in Figure 4.

#### 2.2.1. Single Factor Analysis for Screening of Chelator Concentration Levels

**Figure 5:**
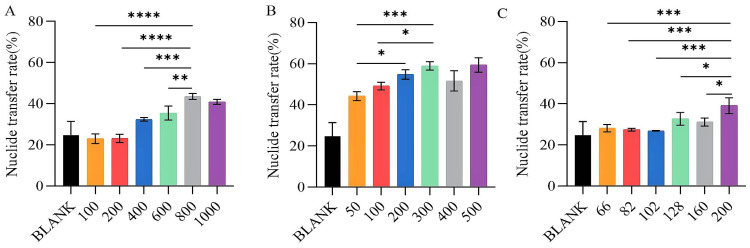
The removal efficiency of uranium of different chelating agents at different concentrations. HOPO (**A**), DFP (**B**), and HEDP (**C**), at different concentrations (µg/mL) on BEAS-2B cells exposed to U(VI). * *p* < 0.05, ** *p* < 0.01, *** *p* < 0.001, **** *p* < 0.0001 is compared to the significant group exposed to U(VI). Bars represent mean ± sd.

This study aims to measure the transfer rates of nuclides to establish the concentration gradients for three distinct chelating agents (Figure 5). These measurements will facilitate the determination of the most effective chelating concentration range for each agent. The data illustrated in Figure 5 demonstrate that as the concentration of the chelating agent increases, the nuclide transfer rate of the nuclide to the exterior of the cell gradually rises. However, when the concentration reaches a specific threshold, the nuclide transfer rate exhibits minimal further change. The nuclide transfer rate of HEDP has been observed to increase due to its low safe concentration. The findings indicate the following optimal concentration ranges: HOPO (700–900 μg/mL) (0.932 mM–1.199 mM), HEDP (150–250 μg/mL) (0.658 mM–1.097 mM), DFP (200–400 μg/mL) (1.097 mM–2.875 mM).

#### 2.2.2. Complexing Ability of Chelating Agents Optimized Using RSM

Box Behnken Design (BBD) is a commonly used response surface design method for optimizing the relationship between multiple variables. It constructs a mathematical model to predict changes in the response variables by selecting specific points within the experimental region to conduct experiments. BBD experiments are widely used in multi-variable optimization experiments by reducing the number of experiments and improving the robustness and predictive capabilities of the model. Its evenly distributed experimental points and three-level design give it significant advantages when handling complex processes [26]. Thus, variables selected for Y2 (X1, X2, and X3) in the implemented BBD of the experiment (Table 1) were identified through initial experiments.

The findings presented in Table 2 were derived using the RSM design tailored to enhance the complexation capabilities, with Y2 serving as the dependent variable. Table 3 illustrates the variance analysis (ANOVA) along with the fitting parameters for the quadratic polynomial model used in the optimization study.

As a result, non-linear equations were formulated using the second-order polynomial model. Subsequently, for Y2,

(1) $$Y2=270.14-4.15A-9.65B+1.53C+9.37AB+1.82AC+0.325BC\;+18.97A^{2}+10{.02B}^{2}-11.58C^{2}$$

**Figure 6:**
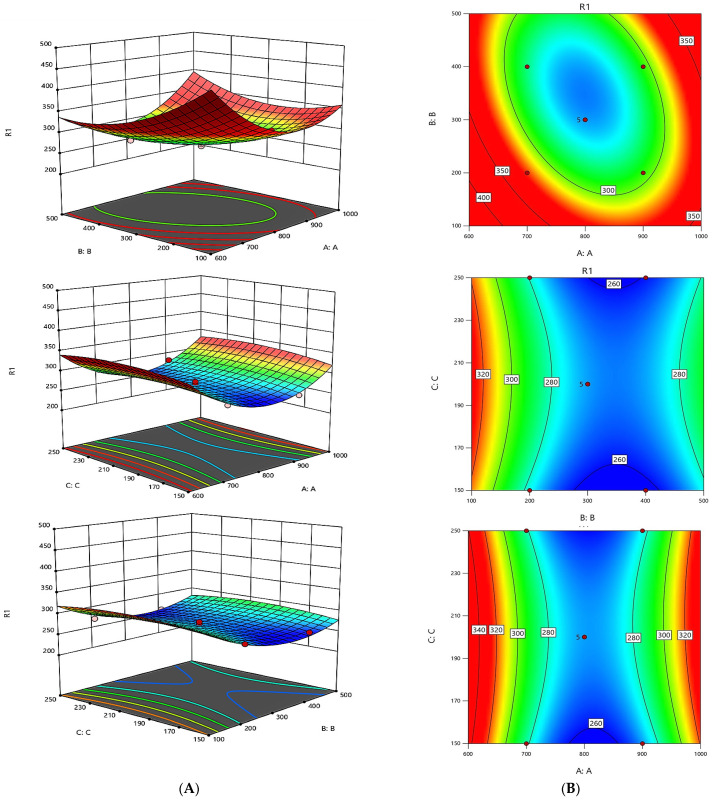
Graphical results in terms of the response surfaces of the format value of Y2. Part (**A**): Joint graphical 3D analysis as a function of each of the variables involved. Each of the net surfaces represents the theoretical three-dimensional response surface predicted with the second-order polynomial as a function of each one of the involved variables. The statistical design and results are described in Table 2. The estimated parametric values are shown in Table 3. Part (**B**): RSM contour charts. From red to blue, the response variable value is from high to low.

The equations previously mentioned elucidate the trends identified in individual data points. Moreover, the pattern profiles that emerge from the parametric evaluations of these equations can be visually illustrated through diagrams. A three-dimensional graphical representation of these patterns for Y2 is displayed in Figure 6.

For the Y2, *p*-values below 0.0500 signify significant terms within the model. Specifically, the terms B, AB, A^2^, B^2^, and C^2^ were marked as significant. No notable interactions were found between A and C or B and C; however, a positive correlation was noted between A and B.

The optimization process effectively minimizes errors by employing a compact experimental design that utilizes few trials. This approach facilitates multivariable fitting, which simplifies the analysis by reducing the number of parameters needed, thereby enhancing the precision of estimates and tightening the confidence intervals. This methodology also improves the predictability of the response behavior. Utilizing a straightforward approach, the ideal conditions for all responses were pinpointed, with a minimum for Y2. The optimal dosages were determined as 824.3 μg/mL (1.10 mM) for HOPO, 343.9 μg/mL (1.10 mM) for DFP, and 249.3 μg/mL (1.09 mM) for HEDP. The conditions that minimize the response of Y2 globally were identified at 259.8 ng/10^4^, establishing an approximate chelating agent ratio of 8:3:2.

### 2.3. Analysis of Survival Rate in Acute Lethal Uranium Inhalation Exposure Model

**Figure 7:**
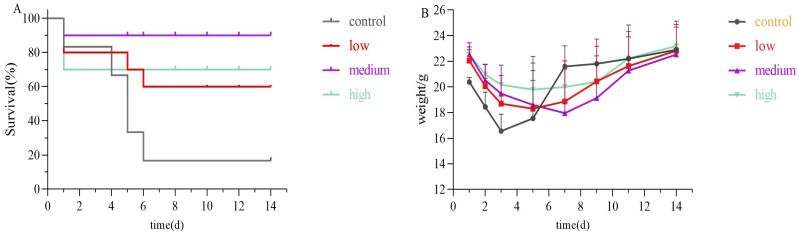
Mice survival rate experiment (**A**) and weight change (**B**) during 14d. Each mice received uranyl nitrate in the lungs and was then either treated promptly or left untreated 10 min after uranium exposure, according to the regimens described above.

Mice were exposed to 80 mg/kg of uranyl nitrate via the tracheal route at a volume of 50 µL to establish an acute inhalation poisoning model. Subsequently, 50 µL of the chelating agent was administered via the tracheal route 10 min after exposure. The control group was administered 50 µL of physiological saline only following exposure. As displayed in Figure 7, during the two-week observation period, the control group saw a survival rate of only 16.7%, with just one animal surviving. However, the combination of chelating agents ‘group exhibited significantly higher survival rates. Specifically, survival rates were 60% in the low-dose group, 90% in the medium-dose group, and 70% in the high-dose group. The improvement in survival rates for the low-dose and medium-dose group demonstrated a dose-dependent relationship. The reduced survival in the high-dose group likely resulted from toxic effects due to the high concentration of agents used.

### 2.4. In Vivo Uranium Removal Capacity

**Figure 8:**
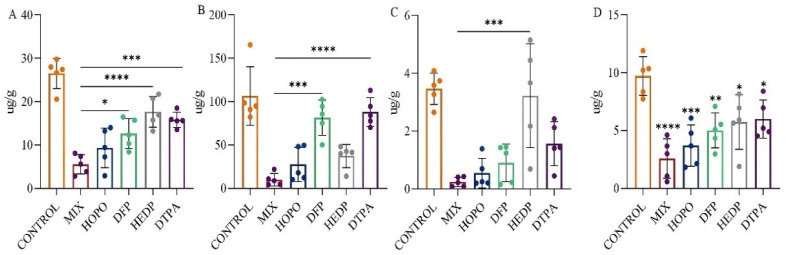
The depletion of uranium in rats was achieved through intratracheal injection after 10 min exposure of single or a combination of chelating agents, with efficacy observed 24 h after exposure. The figure illustrates the uranium content in the tissue (lung (**A**), kidney (**B**), liver (**C**), bone (**D**)). * *p* < 0.05, ** *p* < 0.01, *** *p* < 0.001, **** *p* < 0.0001 is compared to the MIX group (**A**–**C**) or the control group(**D**) exposed to U(VI). Bars represent mean ± sd.

Following the promising results in the survival study, the medium-dose group (HOPO 24 mg/kg + DFP 9 mg/kg + HEDP 6 mg/kg) was chosen for further assessment of their effectiveness. This evaluation involved a comparative analysis with individual chelating agents over a 24 h period to assess their efficacy. Illustrated in Figure 8A–D, a decrease in uranium retention across various tissues was observed for all chelating agent groups when compared to the model control group. This indicates the varying degrees to which different chelating agents can extract uranium from the tissues of affected animals. It was distinctly noted that the group receiving the combination of chelating agents exhibited superior uranium removal compared to groups receiving single agents.

As depicted in Figure 8A, the lung, being the initial organ to encounter uranium via inhalation as per the modeling, experienced a significant reduction in uranium levels with the administration of any chelating agent, as referenced in Table S1 (26.50 ± 3.49 µg/g). Regardless of the type of chelating agent used, all facilitated the rapid clearance of uranium from the lung (*p* < 0.01). Notably, the combination of chelating agents demonstrated the most effective removal, reducing uranium levels to 5.58 ± 2.24 µg/g, which translates to a 79% decrease, a removal rate significantly more effective than that achieved with DTPA, DFP, or HEDP (*p* < 0.01).

As depicted in Figure 8B, the kidney, a critical target organ for uranium toxicity, showed significant reductions in uranium concentrations when treated with the combination of chelating agents. The concentration dropped to 10.27 ± 7.12 µg/g in the combination of chelating agents’ group, achieving a clearance rate of 90%, compared to 106.51 ± 33.54 µg/g in the untreated model control group. Conversely, the clearance performance of DFP and DTPA was comparatively lower, with concentrations remaining at 81.66 ± 20.27 µg/g and 88.00 ± 16.63 µg/g, respectively, indicating less effectiveness in kidney uranium clearance.

Referencing Figure 8C, all chelating agents except HEDP demonstrated efficacy in facilitating the removal of uranium from the liver, with the combination of chelating agents’ group showing the lowest levels of residual uranium. Regarding the bone, as depicted in Figure 8D, the application of any chelating agent expedited the removal of uranium, as evidenced by accelerated reduction rates compared to those in the untreated model control group (9.72 ± 1.67 µg/g) (*p* < 0.05). In both liver and bone, the lowest residual uranium was observed in the combination of chelating agents’ group, illustrating its superior efficacy over single-agent treatments in promoting the excretion of uranium from tissues affected by inhalation.

**Figure 9:**
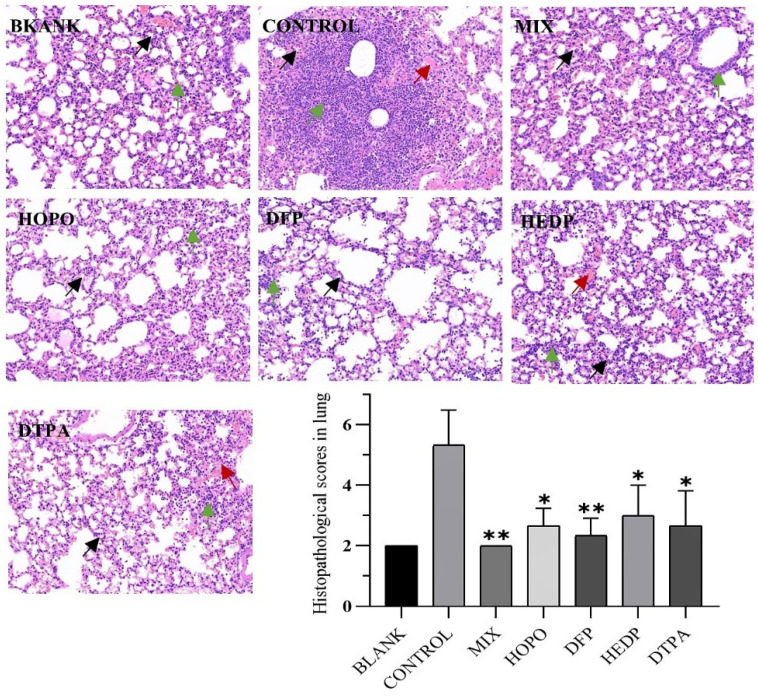
Twenty-four hours following uranyl nitrate poisoning, pathological alterations to lung tissue were observed. Lung and kidney tissue specimens from each group (n = 3) were examined under an optical microscope after HE staining (HE 20×). The experimental groups were identical to those previously described. The significance of the scores (* *p* < 0.05, ** *p* < 0.01) is then compared with the control group. Black arrows, alveolar cavities demonstrated atrophy; green arrow, inflammatory cells infiltrated in the alveolar septum and red arrow, red blood cells observed in alveolar cavities.

In conjunction with the lung pathology results, following inhalation of uranium with no treatment in mice (Figure 9 CONTROL), some alveolar epithelial cells in the lung tissue demonstrated moderate proliferation, the alveolar septum exhibited moderate thickening, a considerable number of alveolar cavities demonstrated moderate atrophy (black arrows), and the tissue displayed slight solidification. The tissue was also observed to have a large number of inflammatory cells focally infiltrated in the alveolar septum (green arrow). Additionally, the tissue exhibited moderate bleeding, with a notable presence of red blood cells observed in some alveolar cavities (red arrow). The timely administration of chelating agents has been demonstrated to improve uranium damage in the short term, effectively improve alveolar atrophy and reduce inflammatory cell infiltration. Furthermore, When the tissue uranium content was combined with the lung pathology score, the MIX group was observed to have no adverse effect on lung damage, indicating its potential for reducing toxicity and increasing efficacy.

## 3. Discussion

The internal environment of the body is complex and changeable. Thus, this article synthesizes and extends the combined in vitro screening and in vivo validation approach. The concurrent utilization of two in vitro techniques (agarose gel and cell models) to assess chelators not only minimizes the number of animals and uncertainty employed for direct screening in animal models [27,28] but also permits a comprehensive evaluation of the complexing capacity of the chelator under identical interference conditions.

Specifically, the agarose gel method [21] provided an effective means of scoring chelators which was suitable for the first screening assessment to predict the chelating ability. This method established a uniform level in vitro to easily evaluate the uranium mobilization ability of different chelators, providing a new idea for establishing an in vitro evaluation model for some soluble chelators. As shown in Figure 2 and Figure 3, all chelators can mobilize uranium excretion, but the ability and rate of mobilizations of different chelators are different. Meanwhile, EDTA and DTPA could only weakly complex uranyl nitrate reported in the literature [12], we found that EDTA and DTPA began to show uranium transfer abilities similar to other chelators at later time points in Figure 2, which was different from the literature. Since we used a relatively excess concentration of chelating agents, most of the uranium can be mobilized over a sufficient period of time, thus reducing the differences in chelating ability among all chelating agents as time progresses. This result is similar to that obtained by Vander Meeren using the agarose gel model. The agarose model is a relatively simple physicochemical model, which is different from the complex situation in vivo and is therefore only suitable for preliminary screening.

Next, the studies at a cellular level can reveal whether the chelator can effectively promote the efflux of uranium. These results are consistent with the studies on the interaction between chelators and nuclides in the literature [29,30,31]. We innovatively used a combination of response surface and cell models to screen chelating agent ratios and develop a novel chelating agent blend comprising 3,4,3-Li (1,2-HOPO), DFP, and HEDP, optimized in a specific ratio (8:3:2).

Finally, we further optimized the ratio and verified its effect in an animal model. This formula not only effectively reduced the retention of uranium in tissues after uranium inhalation, but also alleviated the damage caused by uranium to lung tissue. In this combination of chelating agents, 3,4,3-LI(1,2-HOPO) has been demonstrated to be highly effective against U(VI) due to its strong affinity for hexavalent uranium. It forms stable complexes and enhances urinary excretion. It also has high security [32]. DFP is effective against both Uranium Gallium and Indium toxicity, providing broad-spectrum chelation [33]. HEDP is particularly effective in chelating U(IV) and U(VI), making it useful for a wide range of uranium species. A study [34] shows that both oral and subcutaneous administration of EHBP effectively improves kidney structure and function in animals exposed to lethal oral doses of uranium. In the experiment of Xiaomei Wang et al. [35], the group that received intraperitoneal injection of 5LIO-1-Cm-3,2-HOPO showed reduced accumulation of U(VI) in the kidney and femur, where uranium levels were reduced by 82.9% and 39.0%, The groups given ZnNa3-DTPA showed limited removal of U(VI) under identical experimental conditions (20.5% and 9.1%). In contrast, our formulation achieves similar results (90%) in terms of uranium renal clearance, but with greater efficiency in bone retention (83%). However, due to the different ways of administering uranium and chelating agents, the specific effects need further evaluation.

The rapid removal of uranium at the initial stages of inhalation exposure is paramount [36]. The effectiveness of chelating agents in removing uranium is influenced by several factors, including the chemical properties of the agents and the oxidation state of uranium. Uranium can exist in multiple oxidation states (U(IV), U(V), and U(VI)), each with different chemical behaviors: U(IV): This state is less soluble and tends to form precipitates, making it harder to remove from tissues. U(V): Intermediate in solubility and reactivity, can be relatively easily complexed by chelating agents. U(VI): Highly soluble and reactive, it is the most common form of uranium in environmental and biological systems and is more readily chelated. However, not all chelators are effective in treating the different oxidation states of uranium. The chemical nature of uranium allows it to exhibit a complex behavior in its interactions with different chelating agents. Studies have shown that certain chelators are capable of enhancing the removal efficiency of uranium by forming stable uranium chelates, but this usually depends on the specific oxidation state of uranium [37]; the development of efficient chelators that can adapt to different oxidation states of uranium is an important direction to improve the effectiveness of uranium removal technology.

The combination of chelating agents is designed for early and convenient administration via inhalation and obtains good results in animal models. It effectively increases the survival rate of the acute lethal uranium inhalation exposure mice and reduces uranium levels in their tissues. Similarly, Zandevakili et al. [20] show that the chelator agents with remove Mn from the brain can improve MWM performance. Results indicated that combined therapies were more efficient compared to single therapies. Saric et al. [19] show that zinc concentration was significantly lower in the liver and higher in the kidneys only after Ca-DTPA and combined DMSA and Ca-DTPA chelation.

In scenarios involving exposure to multiple nuclides [38], the combination of chelating agents could offer a wider spectrum of chelation capabilities. In this investigation, 3,4,3-Li (1,2-HOPO), DFP, and HEDP were chosen for their active roles within the combination of chelating formulation. Notably, HEDP has been identified as a potential agent for removing U(VI) and Eu(III) [39,40] while 3,4,3-LI(1,2-HOPO) has shown potential to reduce in vivo retention of Plutonium, Americium, and Uranium [41]. DFP, noted for its lipophilic properties, demonstrated a significant enhancement in cellular excretion [42]. To evaluate the complementary and rational chelating capabilities of these agents towards various nuclides, an agarose gel competitive adsorption assay was utilized, testing each agent at equal concentrations against multiple nuclides. The results (depicted in Figure S1) showed that the three agents varied in their complexation efficiencies for different nuclides. This suggests that the combination may offer broad-spectrum chelating potential. However, further experimental design and verification of the system are still needed though confirmatory in vivo and in vitro studies are necessary to substantiate these findings.

## 4. Materials and Methods

### 4.1. The Efficiency of Radionuclide Transfer in Agarose Gel In Vitro

#### 4.1.1. Actinide Compounds

For the experiments, two uranium compounds were utilized: a soluble compound, uranyl nitrate hexahydrate (UO_2_(NO_3_)_2_ 6H_2_O, Macklin, Shanghai, China), and a less soluble compound, Triuranium octoxide (U_3_O_8_, China). Both compounds, sourced from Macklin, Shanghai, China, with a purity of 99%, were incorporated into the gel matrix at a standardized final concentration of 10 µg/mL.

#### 4.1.2. Preparation of Agarose Gels and Chelators

Agarose solutions were formulated by dissolving agarose (provided by Solarbio, Beijing, China) into physiological saline (NS ratio: 500 mL to 4.5 g, sourced from Kelun, Chengdu, China) to achieve a 2.5% concentration. Five distinct dynamic chelating phases were employed, each adjusted to a pH of approximately 7 and standardized to a final concentration of 400 µg/mL: 3,4,3-Li(1,2-HOPO)(HOPO)(97% purity, lqt-biotech, Chengdu, China), Disodium Etidronate Hydrate (HEDP) (98% purity, Macklin, Shanghai, China), 3-Hydroxy-1,2-dimethyl-4(1H)-pyridone (DFP) (98% purity, Aladdin, Shanghai, China), Penta sodium salt of Diethylenetriaminepentaacetic acid (DTPA-5Na) (98% purity, Macklin, Shanghai, China, 50% aqueous solution), and EDTA disodium salt dehydrate (EDTA) (99% purity, Biofroxx, Guangzhou, China).

The agarose solution was liquefied by heating on an induction cooker and allowed to cool at ambient temperature for 5 min before the addition of the actinide under mild agitation. Subsequently, this mixture was allocated into 24-well culture dishes at 300 µL per well. Once the gel solidified, representing the static phase, 700 µL of the dynamic phase was introduced into each well. The experimental setup included maintaining the plates at 37 °C on an orbital shaker within a constant temperature oscillator to ensure even distribution of nuclides and prevent sedimentation. Replicates were conducted in triplicate for all experimental variables. Control experiments were conducted using contaminants in gels that were prepared in saline and incubated with a dynamic phase also consisting of saline.

#### 4.1.3. Collection of Fluids and Content Measurement

Samples of the dynamic phase (100 µL) were systematically collected at designated intervals (5 min, 15 min, 30 min, 1 h, and 2 h), and an equivalent volume was promptly replenished to maintain consistent conditions within the wells. Following the removal of the gel from each well, the retrieved samples underwent digestion prior to the quantification of their nuclide concentrations using Inductively Coupled Plasma Mass Spectrometry (ICP-MS, Model 7900, Agilent, Santa Clara, CA, USA).

### 4.2. The Chelating Agent Ratio of BEAS-2B Cell Based on the Response Surface Model

#### 4.2.1. Cell Line and Culture

The BEAS-2B cell line is a human normal lung epithelial cell strain, provided by Servicebio (Wuhan, China), was maintained in a growth medium consisting of DMEM (supplied by gibco, Suzhou, China), supplemented with 10% fetal bovine serum (provided by Cytiva, Shanghai, China) and 1% Penicillin-Streptomycin solution (Pricella, Gibco, Wuhan, China). Cultivation took place in an environment with a humidified atmosphere containing 5% CO_2_ at 37 °C. The cells were subcultured bi-daily using a 0.25% trypsin solution (Sigma, Cream Ridge, NJ, USA).

#### 4.2.2. Initial Screening of Concentration Range for Chelating Agents and Uranyl Nitrate

Cytotoxicity evaluations were conducted on both chelating agents and uranyl nitrate to ascertain their viable concentrations for use in U(VI) removal studies. In order to ensure a safe concentration for each chelating agent, in vitro cytotoxicity screening was conducted with the protocol of the international standard norm ISO 10993 [43]. The cytotoxicity experiment can be carried out as follows:

Prepare chelating agent solutions at different concentrations and set up a blank control group (add only blank medium).

For the chelating agents: take logarithmic phase BEAS-2B cells, digest and centrifuge the cells, then resuspend and count them. Seed 3000 cells per well in a 96-well plate, with 6 replicate wells for each concentration. Incubate the cells in a culture incubator for 24 h. Remove the supernatant from the 96-well plate and add 100 µL of the different concentrations of chelating agents to each well. Incubate the cells with the chelating agents for 24 h. Add 10 µL of CCK-8 (Beyotime, GK10001, Nantong, China) reagent to each well and continue incubating at 37 °C in the dark for 2 h. Measure the absorbance of each well at 450 nm using a microplate reader.

For the uranyl nitrate: As the concentration of uranyl nitrate increases, it tends to form a precipitate that interferes with the absorbance measurement. So, we chose the MTT to screen the concentration range of uranyl nitrate. After the incubation period, remove the supernatant and add 100 µL of MTT (Solarbio, M8180, Beijing, China) solution to each well. Continue incubating at 37 °C in the dark for 4 h. Remove the MTT solution and add 100 µL of DMSO (Innochem, NBCZBIIB, Beijing, China) to each well. Measure the absorbance of each well at 490 nm using a microplate reader.

#### 4.2.3. Quantification of Chelating Agents

For the single-factor study, concentrations were selected based on the cytotoxicity experiment for HOPO (ranging from 100 to 1000 μg/mL), DFP (from 50 to 500 μg/mL), and HEDP (from 65.54 to 200 μg/mL). The quantification of chelating agents is as follows:

Logarithmic phase BEAS-2B cells were taken, seeded at 2 × 10^5^ cells per mL in each well of 6-well plates, were exposed to uranyl nitrate at a concentration of 20 μg/mL and cultured for 24 h. Subsequently, the U solutions were discarded, and the wells were rinsed with Phosphate Buffered Saline (PBS) (Gibco, Suzhou, China). Cells then received 1 mL of medium containing varied concentrations of the selected chelating agents and were incubated for 8 h at 37 °C. Post-incubation, solutions from each well were transferred into tubes, and the wells were again rinsed with PBS. Cell lysis was conducted using 70% concentrated nitric acid (400 μL, repeated twice), with the lysates collected for analysis. Uranium levels in both supernatants and cell lysates were quantified using ICP-MS. These procedures were replicated three times for accuracy.

#### 4.2.4. Box–Behnken Model Study

Utilizing the concentrations of HOPO (X1), DFP (X2), and HEDP (X3) in µg/mL as individual factors, we determined the optimal combination of these chelating agents using the Box–Behnken design approach. The response variable X, shown in Table 1, was analyzed to establish the most effective ratios for chelation. The specific steps are as follows:

To conduct the experiment, first, prepare chelating agent solutions in a blank culture medium at specified concentrations: HOPO at 700 μg/mL, 800 μg/mL, and 900 μg/mL; DFP at 200 μg/mL, 300 μg/mL, and 400 μg/mL; and HEDP at 150 μg/mL, 200 μg/mL, and 250 μg/mL. Following preparation, group and mix these solutions as indicated in Table 2 for further use.

Next, inoculate cells in the logarithmic growth phase into a 6-well plate, with 2×10^5^ cells per well, and incubate for 24 h to allow cell attachment and initial growth. Afterward, introduce uranium poisoning by adding 1 mL of 20 μg/mL uranyl nitrate (prepared in blank culture medium) to each well, continuing incubation for another 24 h to facilitate uranium internalization by the cells. Discard the supernatant and wash the cells twice with PBS to remove uninternalized uranium ions, then add 1 mL of the prepared chelating agent solution according to the mixing instructions from Table 2, and incubate for 8 h to enable chelation of uranium ions. Post-chelation, collect the supernatant into centrifuge tubes, trypsinize the cells, and count them under a microscope. The collected culture medium and cells are to be digested with 70% nitric acid.

Finally, measure the amount of remaining uranyl nitrate’s quantity in the cell sample using ICP-MS.

### 4.3. In Vivo Study

#### 4.3.1. Animal Selection, Preparation of Uranium Solution, and Chelating Agent

Our study utilized non-fasting Balb-c mice, each weighing between 20 and 22 g, sourced from Vital River Laboratory Animal Technology, China. Ethical approval for all experimental procedures was granted by the Ethics Committee of the Beijing Institute of Radiation Medicine (Approval No. IACUC-DWZX-2024-P626), ensuring adherence to the principles of animal welfare. Solutions of uranyl nitrate were prepared in normal saline. Similarly, chelating agents were dissolved in saline and readied for administration. The chelating agents were administered to the mice 10 min following their exposure to the uranium salt solution.

#### 4.3.2. Survival Rate of Acute Lethal Uranium Inhalation Exposure Model

Administered under 1% sodium pentobarbital anesthesia (Isoflurane, Qingdao, China), mice were subjected to an acute exposure model using 80 mg/kg of uranyl nitrate delivered via the trachea in a volume of 50 µL. After a 10 min interval, a 50 µL dose of chelating agent was administered intratracheally to the mice (n = 10 per group). For the control group, only the uranyl nitrate at 80 mg/kg in 50 µL was administered (n = 6). Over the following 14 days, the health metrics including weight changes and mortality were systematically recorded for all groups.

The formula group design: Low Dose (HOPO 8 mg/kg + DFP 3 mg/kg + HEDP 2 mg/kg); Medium Dose (HOPO 24 mg/kg + DFP 9 mg/kg + HEDP 6 mg/kg); High Dose (HOPO 72 mg/kg + DFP 27 mg/kg + HEDP 18 mg/kg).

#### 4.3.3. Tissue Clearance Role in Acute Uranium Inhalation Mouse Model

Administered with 1% sodium pentobarbital for sedation, mice were administered 50 µL of uranyl nitrate at a dosage of 10 mg/kg through the trachea. Ten minutes post-exposure with uranium, a 50 µL dose of a specified chelator was given. At 24 h post-treatment, the mice were humanely euthanized, and tissues including the lungs, liver, kidneys, and right femur were harvested (n = 5 per group). The experimental groups were defined as follows: Group CONTROL received a control treatment of 0.9% normal saline; Group MIX was treated with a mixture of chelators (HOPO at 24 mg/kg, DFP at 9 mg/kg, HEDP at 6 mg/kg); Group HOPO received HOPO at 24 mg/kg; Group DFP received DFP at 9 mg/kg; Group HEDP received HEDP at 6 mg/kg; and Group DTPA received DTPA at 75 mg/kg. The blank group was given saline twice.

### 4.4. Data Presentation and Analyses

Statistical evaluations and the generation of graphical representations were performed using GraphPad Prism version 8.0.1 and SPSS version 26.0. Comparisons among multiple groups were made through one-way ANOVA and Kruskal–Wallis tests, with significance set at *p* < 0.05.

In the context of the agarose gel experiments, the percentage of nuclide transfer was calculated by comparing the radionuclide content in the mobile phase at each time point to its total content in the respective well. The graphs display average values as bars, with standard deviation (SD) depicted through error bars.

In the cellular assays, we evaluated the complexation efficiency by measuring the rate at which uranyl nitrate was transferred from the cells to the external environment (expressed as Nuclide Transfer Rate (%), Y1), along with the residual quantity of uranyl nitrate remaining within the cells (ng/10^4^, Y2).

(2) $$Y1=\frac{S1}{S1+S2}\times 100\%$$

In this model, Y1 represents the percentage of nuclide transfer rate; S1 quantifies the amount of uranium present in the supernatant (ng); and S2 quantifies the uranium content within the cell lysates (ng).

(3) $$Y2=\frac{S2}{S3/10^{4}}$$

where Y2 is the amount of remaining uranyl nitrate’s quantity in the cell (ng/10^4^); S2 defines the uranium in the cell lysates (ng); S3 defines the cell count.

The uranium analysis of animal tissue was conducted using nitration followed by measurement via ICP-MS. The calculation method employed was the ratio of the uranium content in the tissue to the tissue weight (µg/g). The lung specimens were fixed with 4% formaldehyde and, once in a suitable condition, pathological sections were prepared in accordance with standard procedures. The sectioned specimens were stained with hematoxylin and eosin (HE) and examined under an optical microscope. The lesion score was quantified on a scale of 0 to 4 points according to the severity of the lesions observed, namely inflammation, bleeding, and cell proliferation, in each part. A score of 1 indicated mild severity, 2 indicated moderate severity, 3 indicated severe severity, 4 indicated extremely severe severity, and 0 indicated that the tissue was basically normal.

## 5. Conclusions

In conclusion, this study developed a combination of chelating agents composed of 3,4,3-Li (1,2-HOPO), DFP, and HEDP, optimized to a specific ratio of 8:3:2. This formulation was derived through in vitro agarose gel dynamics screening experiments and response surface modeling aimed at enhancing uranium removal efficiency. Subsequent in vivo tests confirmed the agent’s substantial effectiveness in removing inhaled uranium and suggested its potential for broader application in the extraction of other actinides. This indicates that the combination of these agents could serve as a versatile and effective new strategy in the development of chelating agents for nuclide removal, warranting further exploration.

## Figures and Tables

**Table 1:** Group design.

Factor	Level		
X1	700	800	900
X2	200	300	400
X3	150	200	250

**Table 2:** Experimental design and results of response surface.

Std	Run	Types of Chelating Agents			Response
		HOPO (μg/mL)	DFP (μg/mL)	HEDP (μg/mL)	Y2 (ng/10^4^)
1	9	700	200	200	325.3
2	13	900	200	200	300.0
3	7	700	400	200	279.5
4	1	900	400	200	291.7
5	12	700	300	150	278.0
6	4	900	300	150	264.3
7	6	700	300	250	287.1
8	10	900	300	250	280.7
9	15	800	200	150	278.0
10	11	800	400	150	265.8
11	16	800	200	250	270.7
12	2	800	400	250	259.8
13	14	800	300	200	263.5
14	8	800	300	200	271.9
15	3	800	300	200	276.8
16	17	800	300	200	269.9
17	5	800	300	200	268.6

**Table 3:** Analysis of variance (ANOVA) and fitting parameters of the quadratic polynomial model for the optimization process.

Source	SS	DF	MS	F-Value	*p*-Value
Model	3715.01	9	412.78	7.08	0.0086
A-HOPO	137.78	1	137.78	2.36	0.1682
B-DFP	744.98	1	744.98	12.77	0.0091
C-HEDP	18.61	1	18.61	0.3189	0.5899
AB	351.56	1	351.56	6.03	0.0438
AC	13.32	1	13.32	0.2283	0.6473
BC	0.4225	1	0.4225	0.0072	0.9346
A^2^	1514.8	1	1514.8	25.96	0.0014
B^2^	422.53	1	422.53	7.24	0.0310
C^2^	564.86	1	564.86	9.68	0.0170
Residual	408.4	7	58.34		
Lack of Fit	314.43	3	104.81	4.46	0.0913
Pure Error	93.97	4	23.49		
Cor Total	4123.42	16			

SS: Sum of squares, DF: degree of freedom, MS: mean square. Significance level = *p* < 0.05.

## Data Availability

Data are contained within the article.

## Supplementary Materials

The following supporting information can be downloaded at: https://www.mdpi.com/article/10.3390/molecules29235759/s1. Table S1: Removal of uranium nitrate in mice treated by inhalation of different chelating agents at 1 day after uranium exposure.; Figure S1: Competitive adsorption test of equal concentration chelating agents on different types of nuclides.
