# Supplementary material for: Preparation and Evaluation of a Combination of Chelating Agents for the Removal of Inhaled Uranium
Source: Molecules. 2024 Dec 5;29(23):5759. doi: 10.3390/molecules29235759 (PMC11643969; doi:10.3390/molecules29235759)
Supplement: Supplementary file 1 [file molecules-29-05759-s001.zip › molecules-3338749-supplementary.pdf]

Table S1 Removal of uranium nitrate in mice treated by inhalation of different chelating agents at 1 day after uranium exposure. Each mice received uranium nitrate (10 mg/kg) in the lungs, and was then either treated promptly or left untreated at 10min after uranium exposure, according to the regimens described above. Inhibition (%) = percentage uranium content inhibition relative to no treatment. $^{a}p < 0.05$ versus Control; $^{b}p < 0.05$ versus HOPO; $^{c}p < 0.05$ versus DFP; $^{\alpha}p < 0.05$ versus HEDP; $^{\beta}p < 0.05$ versus DTPA.

Prompt (10 min) inhalation treatment		Tissue retention at 1 day after uranium exposure							
		Lung		Liver		Bone		kidney	
Chelator	Dose (mg/kg)	Content (μg/g)	Inhibition (%)	Content (μg/g)	Inhibition (%)	Content (μg/g)	Inhibition (%)	Content (μg/g)	Inhibitio n (%)
Control	-	26.50±3.49	-	3.47±0.55	-	9.72±1.67	-	106.51±33.54	-
Mix	HOPO24 DFP 9 HEDP 6	5.58±2.24	79acαβ	0.25±0.17	93aα	2.59±1.72	73a	10.27±7.12	90acβ
HOPO	24	9.38±4.55	65aαβ	0.55±0.51	84aα	3.70±1.78	62a	27.74±19.64	74acβ
DFP	9	12.66±3.44	52a	0.90±0.65	74aα	5.02±1.52	48a	81.66±20.27	23
HEDP	6	17.67±3.53	33a	3.23±1.80	7	5.72±2.35	41a	37.38±13.35	65aβ
DTPA	75	15.84±1.76	40a	1.57±0.76	55a	6.00±1.64	38a	88.00±16.63	17

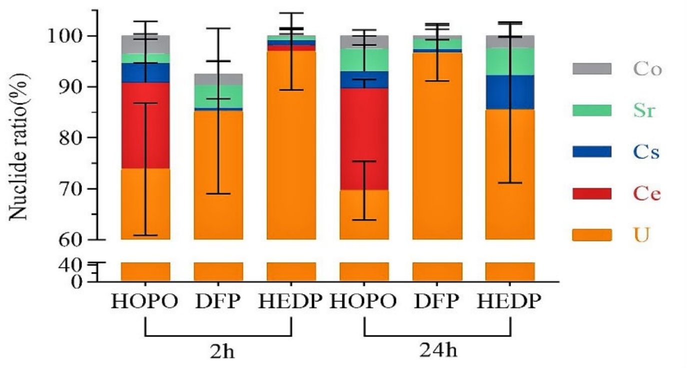

Figure S1. Competitive adsorption test of equal concentration chelating agents on different types of nuclides. Take $UO_2(NO_3)_2{\cdot}6H_2O$ (99%, Macklin, Shanghai, China), Ceric nitrate (99.9%, Macklin, Shanghai, China), Cesium sulfate (99.5%, Macklin, Shanghai, China), Cobalt(III) sulfate hydrate (99.998%, Macklin, Shanghai, China), Strontium acetate (99%, Macklin, Shanghai, China) and add them to normal saline to make the final concentration was 0.5mg/mL. Then distribute the agarose solution into 24-well culture dishes (300μL/well). When the gel hardens, it represents the static phase. Take 700μL of different chelating agents (400 μg/mL) and add it to the well containing gel. Place the 24-well plate in a constant temperature water bath oscillator (50r; 37°C) to shake to prevent the precipitation of nuclides. Each experiment is repeated 6 times. At 2h, collect a 100μL sample from each well and add an equal amount of the same chelating agent to replace it. At 24h, collect 100 μL sample and gel from each well. In each experiment, the control group is represented by normal saline without chelating agents. The samples were measured using ICP-MS. The results are expressed as a nuclides ratio, calculated as follows: (the content of a nuclide in the mobile phase of the chelating agent – the content of a nuclide in the mobile phase of the blank group) / (the content of the mobile phase of the five nuclides of the chelating agent – the sum of the contents of the mobile phase of the five nuclides in the blank group) x100%. In the event of a negative number resulting from the subtraction of data, it is replaced by zero. Each experimental condition is repeated six times. Bars represent mean ± sd.
